# Current trends in regenerative liver surgery: Novel clinical strategies and experimental approaches

**DOI:** 10.3389/fsurg.2022.903825

**Published:** 2022-09-07

**Authors:** Jan Heil, Marc Schiesser, Erik Schadde

**Affiliations:** ^1^ Institute of Physiology, University of Zurich, Zurich, Switzerland; ^2^ Department of General, Visceral, Transplant and Thoracic Surgery, University Hospital, Goethe University Frankfurt, Frankfurt, Germany; ^3^ Chirurgisches Zentrum Zürich (CZZ), Klinik Hirslanden Zurich, Zurich, Switzerland; ^4^ Chirurgie Zentrum Zentralschweiz (CZZ), Hirslanden St. Anna, Lucerne, Switzerland; ^5^ Department of Surgery, Rush University Medical Center Chicago, Chicago, IL, United States

**Keywords:** regenerative liver surgery, future liver remnant, portal vein embolization, ALPPS, liver venous deprivation, simultaneous portal and hepatic vein embolization, resectability

## Abstract

Liver resections are performed to cure patients with hepatobiliary malignancies and metastases to the liver. However, only a small proportion of patients is resectable, largely because only up to 70% of liver tissue is expendable in a resection. If larger resections are performed, there is a risk of post-hepatectomy liver failure. Regenerative liver surgery addresses this limitation by increasing the future liver remnant to an appropriate size before resection. Since the 1980s, this surgery has evolved from portal vein embolization (PVE) to a multiplicity of methods. This review presents an overview of the available methods and their advantages and disadvantages. The first use of PVE was in patients with large hepatocellular carcinomas. The increase in liver volume induced by PVE equals that of portal vein ligation, but both result only in a moderate volume increase. While awaiting sufficient liver growth, 20%–40% of patients fail to achieve resection, mostly due to the progression of disease. The MD Anderson Cancer Centre group improved the PVE methodology by adding segment 4 embolization (“high-quality PVE”) and demonstrated that oncological results were better than non-surgical approaches in this previously unresectable patient population. In 2012, a novel method of liver regeneration was proposed and called Associating Liver Partition and Portal vein ligation for Staged hepatectomy (ALPPS). ALPPS accelerated liver regeneration by a factor of 2–3 and increased the resection rate to 95%–100%. However, ALPPS fell short of expectations due to a high mortality rate and a limited utility only in highly selected patients. Accelerated liver regeneration, however, was there to stay. This is evident in the multiplicity of ALPPS modifications like radiofrequency or partial ALPPS. Overall, rapid liver regeneration allowed an expansion of resectability with increased perioperative risk. But, a standardized low-risk approach to rapid hypertrophy has been missing and the techniques used and in use depend on local expertise and preference. Recently, however, simultaneous portal and hepatic vein embolization (PVE/HVE) appears to offer both rapid hypertrophy and no increased clinical risk. While prospective randomized comparisons are underway, PVE/HVE has the potential to become the future gold standard.

## Introduction

The most common hepatic tumors are liver metastases from colorectal cancer (CRC) (1), which is the third most frequent cancer worldwide (2). Approximately 50% of patients with CRC develop colorectal liver metastases (CRLM) (2, 3). In comparison, primary hepatobiliary tumors are less prevalent and hepatocellular carcinoma (HCC) accounts for 80% of them. HCC is the third most common cause for cancer-related mortality worldwide (2). While liver resection may cure patients with liver tumors, most patients are unresectable (4, 5).

When resectability is assessed, a risk–benefit analysis has to be performed. The amount of liver directly impacts the risk for the patient. The Sloan–Kettering group showed in a landmark paper (6) that the number of resected liver segments correlates with post-operative morbidity and mortality. Besides blood loss, the number of resected liver segment was shown to be the main predictor for post-operative morbidity and mortality, more so than the complexity of the procedure itself (bile duct reconstruction, etc.). With increased usage of preoperative computed tomography (CT) and magnetic resonance imaging (MRI) in the 1990s, analyses shifted from the liver *segments resected* to the planned *liver volume left behind* (7) and from the risk assessment endpoint *complications and mortality* to *post-hepatectomy liver failure* (PHLF). PHLF is to be absolutely avoided in liver resection. For a more accurate estimation of the amount of liver volume left behind, the MD Anderson group introduced the concept of standardized future liver volume (sFLR), i.e., the ratio of the manually measured future liver remnant (FLR) volume to the estimated total liver volume based on biometric formulas (8). Established in meticulous retrospective studies, a minimal sFLR of 25% in healthy livers up to 30% in patients with damaged livers (steatosis and chemotherapy-damaged livers) became the universally accepted cut-off for relatively safe liver surgery, a substantial progress in the field (7, 9–11). Cut-offs for cirrhosis remain controversial.

In patients with a too small sFLR at risk of PHLF, various interventions can be performed that allow an increase in the volume of the sFLR to a larger size prior to resection (12). This article gives an overview of the development and currently utilized strategies in regenerative liver surgery in the face of an increasingly older and comorbid patient population with metastatic liver disease that under no circumstances can be exposed to the risk of PHLF.

## Historical development of regenerative liver surgery

All procedures used to increase liver volume prior to resection make use of the same principle: re-rerouting of portal vein blood to the small future liver (12). Interestingly, this method was already described 100 years ago in an experimental rabbit model by Peyton Rous the Nobel recipient for his work on oncogenic viruses (13). Rous observed in rabbits that the occlusion of the portal branches of a hemiliver results in an atrophy of the occluded main liver and a compensatory hypertrophy of the non-occluded caudate lobe. Sixty years later, Japanese surgeons applied this principle to humans by performing transcutaneous interventional embolization of the portal vein to patients with primary liver cancer and called it portal vein embolization (PVE) (14). While PVE was increasingly used in all types of liver tumors, including metastases (15), a novel concept to reduce the risk of PHLF after the resection of bilobar liver metastasis was introduced by the Paul-Brousse group in 1999, where the resection was performed in two distinct stages and called *two-stage hepatectomy* (TSH) (16).

These two major innovations of the 1990s, PVE and TSH, soon spawned a variety of procedures to improve the surgical treatment of patients with metastatic disease. A foundational review categorizes them into four types (17): (1) The *right first approach* as pioneered by Adam et al. (16), where mostly the main tumor mass is resected during the first stage. PVE can then be performed if necessary (only 6 of 16 patients in the initial series needed it) and the second stage is not performed until several months later (median of 4 months [range 2–14)] in the initial series in order to give the patient and the liver time to recover. (2) The *left first approach* pioneered by the Beaujon group (18), where the left hemiliver is cleaned of tumor in parenchymal sparing resections, followed by a right-sided PVL (in all patients in the initial series), followed rather rapidly by a right or extended right hepatectomy (after a median of 6 weeks, range 4–8). (3) The *left first approach* (19) with PVE between stages was pioneered by the Strasbourg group. Both, PVE and PVL, appear to be equivalent in terms of liver growth (20) and have increasingly been used in metastasis to the liver and hepatobiliary malignancies in the last few decades (21). While PVL cannot be improved much, PVE has been modified by the MD Anderson group to include segment 4 embolization (“high-quality PVE”) in patients with the need for an extended right hepatectomy (22). Also, the prospective randomized “BestFLR” trial showed the superiority of *n*-butyl-cyanoacrylate to other embolic agents in terms of liver growth (23). (4) The *in-situ-split hepatectomy* technique was introduced in 2012 by the Regensburg group (24) to accelerate liver growth between stages by performing an additional transection of the liver parenchyma in addition to PVL. Investigations into the physiological mechanisms in animals revealed that the transection inhibits the formation of portal vein collaterals between the portalized and the deportalized liver lobe, which decreases the portal hyperflow to the FLR and the steal of hepatotrophic factors from the growing FLR (25).

**Figure 1:**
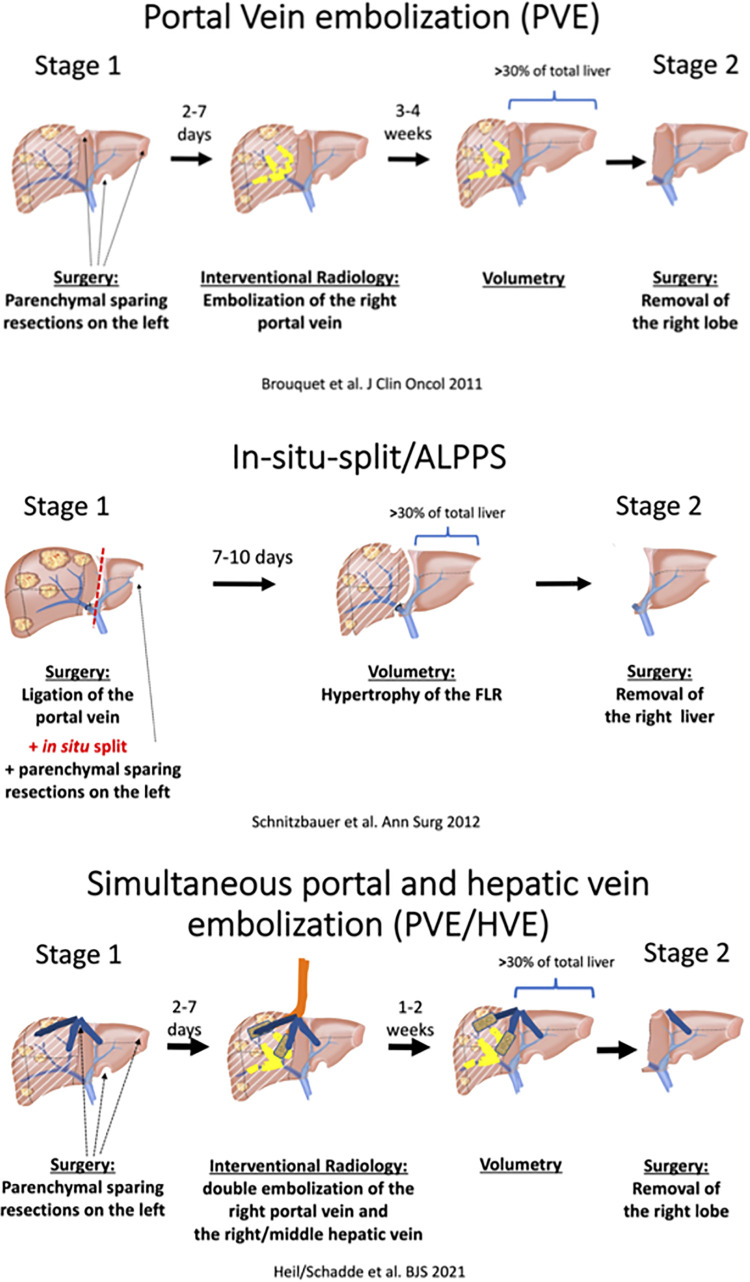
Type of procedures.

In addition to these four regenerative procedures, a fifth variant was introduced in 2016, when the Montpellier group proposed the liver venous deprivation technique (LVD) by abrogating portal venous collaterals by adding a hepatic vein occlusion to PVE in a single interventional procedure (26). This procedure was later simplified as “PVE/HVE” or double embolization (Figure 1).

## The new paradigm of rapid hypertrophy: ALPPS

Re-routing of portal vein flow induces liver volume growth but not to the same extent as the rapid regenerative growth after major hepatectomy (27). However, the capacity of the liver to grow *rapidly* after portal vein rerouting without tissue removal was discovered by chance in 2007, when PVL was combined with an *in-situ-split* of the liver parenchyma by Hans Schlitt in Regensburg, Germany (28). Massive volume gain was observed fortuitously on a CT scan one week after the procedure that had been planned as an extended liver resection but was aborted due a small FLR. The rapid growth then made the resection possible. Schnitzbauer et al. (24) published a prospective series about the *in-situ-split* hepatectomy approach with an impressive percent hypertrophy of 74% (range: 21%–192%) and a curative resection in all patients after a median of 9 days (range: 5–28 days). This new two-stage approach was designed for right trisectorectomies, allowed faster resection, and gave hope to expand the limitations of technical resectability in patients with extensive tumor load. Santibañes and Clavien (29) promoted the procedure under the new name of “*ALPPS*” (Associating Liver Partition and Portal vein ligation for Staged hepatectomy). First reports described an unacceptably high morbidity and mortality risk, but soon modifications of the procedure tried to improve on the early results.

The Scandinavian LIGRO trial from 2018 (30) was the first randomized controlled trial (RCT) comparing ALPPS vs. TSH with PVE/PVL. The trial demonstrated an increased resection rate after ALPPS compared with the conventional techniques (ALPPS: 92% vs. TSH: 57%, *p* < 0.001). A follow-up evaluation also showed oncological superiority of ALPPS, as the higher transection rate in ALPPS translated directly into a significantly better median survival (ALPPS: 46 months vs. TSH: 26 months, *p* = 0.028) (31). However, LIGRO was criticized because of the high number of patients with insufficient liver growth, failure to achieve surgical resection in the control group, and the high mortality in both groups compared with other retrospective reports (32). It was argued that the observed superiority of ALPPS was more or less a result of the poor performance of the control group.

## Limitations of regenerative liver surgery in two stages

The concept of TSH with the addition of regenerative maneuvres became well-accepted in the treatment for liver metastases when showing a comparable long-term overall and disease-free survival compared with one-stage resection despite a higher tumor load (33).

However, liver volume gain induced by PVE/PVL remained limited and high dropout rates up to 30%–43%, mostly due to tumor progression while awaiting sufficient liver growth, remained the Achilles heel of regenerative liver surgery (20, 30, 34). For patients who fail to complete TSH, chemotherapy remains the only treatment with an oncological outcome that is worse than those completing TSH (35).

ALPPS was hailed as a major breakthrough in regenerative liver surgery, as it sparked the hope to overcome these limitations of PVE/PVL (29). However, the initial hype around ALPPS masked a considerable downside of the rapidly induced liver regeneration by a two-stage approach that was actually obvious from the very beginning. Already the initial series by Schnitzbauer et al. reported a high morbidity of 68% and an in-house mortality of 12% (24), but as the procedure spread through hospitals worldwide, many surgeons experienced the high morbidity of the procedure first hand. An early analysis of the worldwide ALPPS registry at the University of Zurich revealed a heterogenous practice pattern of ALPPS regarding indications, selection of patients, and technical modifications (36). Early adopters of ALPPS reported morbidities (major complications) and 90-day mortalities of 44%–75% (37, 38) and 15%–48% (37–40), respectively. The second report of the ALPPS registry (41) revealed age >60 years (odds ratio (OR): 14.3) and hepatobiliary malignancies (OR: 3.1) as independent risk factors for mortality and complications in patients with CRLM. Specifically, in the prospective randomized setting of LIGRO, morbidity (ALPPS: 43% vs. TSH: 43%, *p* = 0.99) and mortality rates remained high (ALPPS: 9% vs. TSH: 11%, *p* = 0.82) (30).

Since PHLF was identified as the leading cause of post-operative mortality despite a sufficient liver volume gain after ALPPS stage 1, the question was raised if function increases proportionally to volume in the rapidly growing liver (41). Pre-clinical (42, 43) and clinical studies (44) from the Amsterdam group using technetium-99m (^99m^Tc) mebrofenin hepatobiliary scintigraphy (HBS) supported the hypothesis of an immature liver after rapid liver growth. A multicentric study by the Amsterdam group revealed an overestimation of liver function by a factor of 2.9 compared with volume after ALPPS stage 1 (44). However, in contrast to these findings, a more recent series showed that function actually increased more (2.8-fold) than volume in ALPPS (*p* = 0.009) (45). In any case, liver volume increase appeared not to be a reliable indicator of liver function in the rapidly growing liver and sparked a renewed interest in liver function assessment. The Amsterdam group proposed an uptake ratio of >2.7%/min/m^2^ in HBS scanning as cut-off for safe liver resection (46). Also, further series confirmed that this cut-off was more reliable than volume to predict PHLF (45, 47), regardless of histological damage and laboratory liver function parameters. Nevertheless, HBS is not widely available across many HPB centres, most likely due to specific know-how required and costs incurred by the procedure.

In summary, ALPPS was hailed as a major breakthrough in regenerative liver surgery (29). But after an initial hype, ALPPS turned out to be too complex and dangerous to replace TSH with PVE or PVL (48–50). Dragged down by a low safety profile and limited by the concept of a two-stage procedure, it is not a versatile enough strategy in an aging patient population and to also be used for primary liver tumors like HCC and cholangiocarcinoma. Nevertheless, ALPPS demonstrated the advantages of rapid hypertrophy to improve resectability and survival in metastatic liver tumors and paved the way for the concept of *rapid hypertrophy*.

## ALPPS modifications

Driven by the allure of *rapid hypertrophy*, a variety of modifications were introduced to improve the safety of ALPPS (Figure 3).

**Figure 2:**
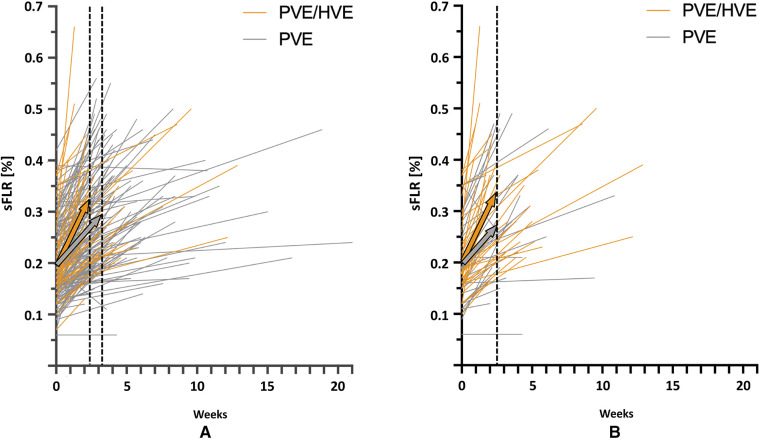
Recent innovations in rapid regenerative liver surgery.

Surgical severity was reduced by choosing a minimally invasive approach despite the complexity of the operation. After a first laparoscopic case series in 2012 (51), Machado et al. presented a comparative series of open vs. laparoscopic ALPPS (*lap-ALPPS*) in 2016 (52). In this series, which mostly included patients with CRLM (Table 1), both ALPPS stages were performed entirely laparoscopically. Major complications (>3A Dindo–Clavien) were significantly reduced in lap-ALPPS (*p* = 0.006), while liver growth was comparable. Although an era bias could not be ruled out, the study showed that lap-ALPPS is feasible and safe in patients with CRLM. Further series confirmed the decreased physiological severity of the minimally invasive approach (54, 60, 61). Also, *Robotic-ALPPS* is feasible (62) and was demonstrated in several case series (54, 63).

**Table 1:** ALPPS modifications.

Author/year	Acronym	*N*	Tumor type	Time between the stages (day)	Hypertrophy	Feasibility of resection	Complications (Dindo–Clavien)	Post-hepatectomy liver failure
Machado et al. (52)	lap-ALPPS	ALPPS: 20 vs. lap-ALPPS: 10	ALPPS: CRLM: 17 Other: 3 vs. lap-ALPPS: CRLM: 9 Other: 1	ALPPS: 21 (11–38) vs. lap-ALPPS: 21 (9–30)	ALPPS: 152% (56–215) vs. lap-ALPPS: 118% (42–157)	ALPPS: 18/20 (90%) vs. 10/10 (100%)	>IIIA:^a^ ALPPS: 10/20 (50%) vs. lap-ALPPS: 0/10	ISGLS:^a^ ALPPS: 8 (44%) vs. lap-ALPPS: 0/10
Robles et al. (53)	T-ALPPS	TSH: 21 vs. T-ALPPS: 21	TSH: CRLM: 21 vs. CRLM: 21	TSH: 45 (28–60) vs. T-ALPPS: 15 (9–31)	TSH: 39% (21–66) vs. T-ALPPS: 68% (22–100)	TSH 19/21 (91%) vs. T-ALPPS: 21/21 (100%)	≥IIIB:^b^ TSH: 3/19 (16%) vs. T-ALPPS: 2/21 (10%)	≥IIIB:^b^ TSH: 2/19 (11%) vs. T-ALPPS: 2/21 (10%)
Jiao et al. (54)	RALPPS	TSH: 24 vs. RALLPS: 26	TSH: CRLM: 19 Other: 5 vs. RALPPS CRLM: 20 Other 6	TSH: 35 (21–75) vs. RALPPS: 20 (SD 14–36)	TSH: 18% (±10%) vs. RALPPS: 81% (±14%)	TSH: 16/24 (67%) vs. RALPPS: 24/26 (92%)	≥IIIA:^b^ TSH: 1/16 (6%) vs. RALPPS: 5/24 (21%)	n.r.
Petrowsky et al. (55)	p-ALPPS	ALPPS: 18 vs. p-ALPPS: 6	CRLM: 16 Other: 8	ALPPS: 9 (range 7–69) vs. p-ALPPS: 11 (range 7–21)	ALPPS: 61% vs. p-ALPPS: 60%	ALPPS: 18/18 (100%) vs. p-ALLPS: 6/6 (100%)	≥IIIB:^b^ ALPPS 6/18 (33%) vs. p-ALPPS: 2/6 (33%)	n.r.
Alvarez et al. (56)	p-ALPPS	ALPPS: 9 vs. p-ALPPS: 21	CRLM: 19 Other: 11	ALPPS and p-ALPPS: 6 (range 4–67)	ALPPS: 107% (SD ± 12) vs. p-ALPPS: 90% (SD ± 21)	ALPPS and p-ALPPS: 29/30 (97%)	≥IIIA:^b^ ALPPS and p-ALPPS: 13/29 (45%)	ISGLS:^b^ ALPPS and p-ALPPS 2/29 (7%)
Rassam et al. (45)	p-ALPPS	ALPPS: 12 vs. p-ALPPS: 9	ALPPS: CRLM: 11 Other: 1 vs. p-ALPPS: CRLM: 6 Other: 3	ALPPS: 15 (IQR 10–19) vs. p-ALPPS: 17 (IQR 14–42)	n.r.	ALPPS: 10/12 (83%) vs. p-ALPPS: 6/9 (67%)	≥IIIA:^b^ ALPPS: 3/10 (30%) vs. p-ALPPS: 1/6 (17%)	ISGLS:^b^ ALPPS: 2/10 (20%) vs. p-ALPPS: 0/6
Robles-Campos et al. (57)	Tp-ALPPS	T-ALPPS: 6 vs. Tp-ALPPS: 6	T-ALPPS: CRLM: 6 vs. Tp-ALPPS: CRLM: 6	n.r.	T-ALPPS: 68% (SD 40–97) vs. Tp-ALPPS: 69% (SD 39–99)	T-ALPPS: 6/6 (100%) vs. Tp-ALPPS: 6/6 (100%)	≥IIIB:^b^ T-ALPPS: 1/6 (17%) vs. Tp-ALPPS: 0/6	ISGLS:^b^ T-ALPPS: 2/6 (33%) vs. Tp-ALPPS: 0/6
Santibañes et al. (58)	Mini-ALPPS	4	CRLM: 3 Other: 1	n.r.	61% (range 49–79)	4/4 (100%)	≥IIIA:^b^ 0/4	ISGLS:^b^ 0/4
Li et al. (59)	hALPPS	2	GB: 2	Case 1: 10 Case 2: 15	case 1: 85% case 2: 66%	2/2 (100%)	≥IIIA:^b^ 1/2 (50%)	ISGLS:^b^ 0/2

ALPPS, associating liver partition and portal vein ligation for staged hepatectomy; CRLM, colorectal liver metastasis; GB, gallbladder cancer; hALPPS, hybrid ALPPS; ISGLS, international study group of liver surgery; lap-ALPPS, laparoscopic ALPPS; p-ALPPS, partial-ALPPS; RALPPS, radiofrequency-assisted liver partition with portal vein ligation for staged hepatectomy; TSH, two-stage hepatectomy; T-ALPPS, Tourniquet ALPPS; Tp-ALPPS, Tourniquet partial-ALPPS.

^a^ In both stages.

^b^ Post stage 2.

Others reduced the surgical trauma during open ALPPS stage 1. Robles et al. (64) proposed a tourniquet parenchymal ligation instead of surgical transection in his tourniquet ALPPS modification, *T-ALPPS*. The liver parenchymal was not transected to prevent collateralization but simply ligated with an umbilical tape. In a first series in 2014 (64), mostly in patients with CRLM, liver growth and post-operative outcome appeared to be similar to ALPPS. Subsequently, in a propensity score-matched analysis in patients with CRLM, T-ALPPS was compared with TSH with PVL during stage I (Table 1) (53). T-ALPPS resulted in enhanced liver growth, but disappointingly, there was no difference in terms of major morbidities (≥IIIB Dindo–Clavien) and mortality.

Jiao et al. proposed radiofrequency ablation of the liver parenchyma instead of transection or ligation during a laparoscopically performed stage 1 in his *RALPPS* modification (radiofrequency-assisted liver partition with portal vein ligation for staged hepatectomy) in 2015 (61). The Hammersmith team performed an RCT of RALPPS vs. TSH with PVE in the REBIRTH trial (rapid induction of liver regeneration for major hepatectomy) (54). While complications were comparable, more liver growth (*p* < 0.001) and a higher resection rate (*p* = 0.007) in RALPPS were observed, further supporting the concept that rapid hypertrophy increases resectability.

A third modification to prevent collateralization, partial-ALPPS (*p-ALPPS*) (55), proposed to transect only 50%–80% of the liver parenchyma to maintain the middle hepatic vein to preserve the venous drainage of segment 4. In three comparative series, including various tumor entities (45, 55, 56), p-ALPPS and ALPPS resulted in comparable liver growth (Table 1). One series also provided functional data by HBS (45), showing that function increased significantly more than volume in ALPPS but not in p-ALPPS. The lower increase in function in p-ALPPS was also observed in two further series (56, 60), and this may be explained by the above-mentioned portal vein collaterals that are not entirely abrogated by the incomplete transection (25). In two of three series, major complications did not differ (Table 1) (45, 55), while in one series, p-ALPPS reduced 90-day mortalities compared with ALPPS (55). In a third series (56), complete transection was found to have a significant impact on post-operative complications in ALPPS (odds ratio: 15.7, 95% CI: 1–244, *p* = 0.049). The concept of a partial transection was also investigated in tourniquet ALPPS in a small comparative series of T-ALPPS against “*Tp-ALPPS*” (tourniquet partial-ALPPS) (57). Both approaches displayed no significant difference regarding post-operative outcome and hypertrophy (Table 1). The combination of a partial transection with an intraoperative PVE, which was called “*mini-ALPPS*”, was also presented in a small series (58). No complication occurred and liver growth induced by mini-ALPPS appeared to be similar to ALPPS (Table 1).

Other innovators focused on modification of the portal vein re-routing after parenchymal transection. Inspired by a case of tumor infiltration of the right hilum making classic ALPPS impossible, “hybrid ALPPS” (*hALPPS*) was proposed by the Hamburg group. In hALPPS, PVE replaced PVL of ALPPS and was performed on post-operative day 2 after ALPPS stage 1 (59). The results were reported only in a case series, and therefore conclusions cannot be drawn (Table 1).

According to a report from the ALPPS registry in 2017 (65), modified ALPPS procedures now encompass more than half of all ALPPS procedures performed since 2015. The authors also observe a decrease in complications as the use of modified versions of ALPPS has increased. This, however, may simply reflect changes in patient selection, specifically an increased prevalence of metastatic disease over primary hepatic tumors.

## Simultaneous portal and hepatic vein embolization (PVE/HVE)

In 2016, the Montpellier group described a new modification of PVE that induced liver regeneration as rapid as ALPPS by simultaneous embolization of the ipsilateral hepatic vein, the LVD technique (26). PVE of the right portal vein was performed using *n*-butyl-cyanoacrylate plus iodized oil (NBCA/lipiodol), and simultaneously, Vascular Amplatzer Plugs (AVP, Abbott Vascular, formerly St. Jude Medical) were utilized for outflow, i.e. hepatic vein embolization (HVE). Additionally, NBCA/lipiodol was injected with meticulous precision into small hepatic veins proximal to the AVPs to obstruct potential venous collaterals that became visible during the procedure.

One year later, the Montpellier group added embolization of the middle hepatic vein to the embolization of the right hepatic vein to increase the effect and called it *extended LVD* (eLVD) (66). These findings in humans confirmed the results of studies from the Chicago Rush group in pigs (67) that demonstrated that simultaneous ligation of both portal and ipsilateral hepatic vein (“double ligation”) did not—as expected—result in necrosis of the respective part of the liver as the liver remained viable by arterial perfusion alone. Rather, the double ligation completely abrogated the formation of collaterals from the FLR, which are commonly observed in PVE/PVL, presumably due to the lack of venous outflow, and induced rapid hypertrophy of the liver that is comparable, if not higher than to what can be achieved in the ALPPS model in pigs (25, 67). These findings also argued against the *trauma theory* of rapid hypertrophy that presumes that the hypertrophy effect of ALPPS results from the trauma of the parenchymal transection during the in-situ-split and the respective increase in inflammatory cytokines like interleukine-6 (IL-6) and tumor necrosis factor alpha (TNF-α) that have known pro-proliferative properties (68, 69). The finding that abrogation of collaterals accelerates hypertrophy rather supports the hemodynamic theory that the formation of steal collaterals by transection is what blunts the proliferative effect of PVE and PVL. The importance of steal collaterals is further supported by the findings that the interventional abrogation of large collaterals re-establishes volume growth in cases of failed PVE (70, 71), and that the degree of transection of the parenchyma—and the resulting prevention of collaterals—correlates with the degree of hypertrophy in partial-ALPPS (72).

Six retrospective comparative cohort studies have been published so far to compare simultaneous embolization of the portal and hepatic veins with PVE alone (Table 2) (73–78). In one series, simultaneous portal and hepatic vein embolization was compared with ALPPS (79). Except for the series by the Montpellier group (26, 66, 73, 77) and one series from Bordeaux (76), all other groups decided to forego the additional liquid embolization of the venous system (74, 75, 78), most likely due to the risk of liquid embolization of the pulmonary vein. The Bordeaux group gave their procedure a name different from “LVD” and “eLVD”: “RASPE” (radiological simultaneous portohepatic vein embolization) (76), while using additional liquid embolization like in the original LVD technique by Guiu et al. (26). Others, unfortunately, did not provide sufficiently detailed information about their embolization techniques (74, 79). In order to avoid further confusion, the generic term “*PVE/HVE*” was introduced by us (78, 80) to refer to the simultaneous embolization of the portal and hepatic vein without additional venous liquid embolization.

**Table 2:** Comparative series about PVE/HVE.

Author/year	Study design	*n*	Age (PVE/HVE)	Tumor type (PVE/HVE)	Peri-interventional complications (Dindo–Clavien) (PVE/HVE vs. PVE)	Intervention to first imaging (days) (PVE/HVE vs. PVE)	Percent hypertrophy (PVE/HVE vs. PVE)	Kinetic growth rate (KGR) (PVE/HVE vs. PVE)	Resection rate (PVE/HVE vs. PVE)	Post-operative Complications (Dindo–Clavien) (PVE/HVE vs. PVE)	Post-hepatectomy liver failure (PVE/HVE vs. PVE)
Panaro et al. (73)	Comparative series	PVE/HVE: 13 PVE: 16	n.r.	CRLM: 10 HCC: 3	0 vs. 0	21 vs. 21	n.r.	16 cc/day (SD ± 7) vs.5 cc/day (SD ± 4)	13/13 (100%) vs. 15/16 (94%)	Major ≥ IIIa: 1/13 (8%) vs. 3/15 (20%)	3/13 (23%) vs. 2/15 (13%)
Kobayashi et al. (74)	Comparative series	PVE/HVE: 21 PVE: 39	65 (range 25–85)	CRLM: 10 HCC: 2 PHCC: 8	Minor: 1/21 (5%) vs. Minor: 0	22 (IQR 17–30) vs. 26 (IQR 20–33)	35% (IQR 23%–54%) vs. 24% (IQR 7%–40%)	2.9% FLR/week (IQR 1.9–4.3) vs. 1.4% FLR/week (IQR 0.7–2.1)	20/21 (95%) vs. 30/39 (77%)	Major > III: 7/20 (35%) vs. 11/30 (37%)	n.r.
Le Roy et al. (75)	Comparative series	PVE/HVE: 31 PVE: 41	65 (CI 55–70)	CRLM: 18 HCC: 5 IHCC: 2 PHCC: 5 Other: 1	0 vs. 0	26 vs. 27	51.2% (SD ± 41.7%) vs. 31.9% (SD ± 34%)	19% FLR/week (SD ± 18) vs. 8% FLR/week (SD ± 13)	25/31 (81%) vs. 31/41 (76%)	Major > IIIa: 5/25 (20%) vs. 3/31 (10%)	n.r.
Laurent et al. (76)	Comparative series	PVE/HVE: 37 PVE: 36	64 (IQR 61–71)	CRLM: 23 IHCC: 7 HCC: 4 NET: 2	Minor: 37/37 (100%) vs. Minor: 34/36 (94%)	31 (IQR 21–40) vs. 30 (IQR 25–43)	61% (range 18–201) vs. 29% (range 9–61)	n.r.	32/37 (86%) vs. 32/36 (89%)	Major ≥ IIIa: 6/32 (19%) vs. 10/32 (31%)	ISGLS: 0/32 vs. 7/32 (22%)
Guiu et al. (77)	Comparative series	PVE/HVE: 29 PVE: 22	62 (IQR 26–79)	Metastases: 22 IHCC: 4 HCC: 2 Other: 1	Minor: 6/29 (21%) vs. Minor 3/22 (14%)	21 vs. 21	53% (min–max: 1–176) vs. 19% (min–max: 11–102)	n.r.	21/22 (96%) vs. 27/29 (93%)	Major ≥ IIIa: 3/21 (14%) vs. 3/27 (11%)	50–50 criteria: 0 /21 vs. 0/27
Heil et al. (78)	Comparative series	PVE/HVE: 39 PVE: 160	63 (IQR 52–67)	CRLM: 19 HCC: 4 IHCC: 4 PHCC: 5 GBC: 4 Other: 3	Minor: 5/39 (13%) Major: 1/39 (3%) vs. Minor: 22/160 (14%) Major: 3/160 (2%)	17 (IQR 13–32) vs. 24 (IQR 19–37)	59% (IQR 45–79) vs. 48% (IQR 24–69)	3.5% sFLR/week (IQR 2.2–7.1) vs. 2.5% sFLR/week (IQR 1.1–3.8)	35/39 (90%) vs. 109/160 (68%)	Major > IIIA: 9/35 (26%) vs. 37/109 (34%)	ISGLS: 4/35 (11%) vs. 27/109 (25%)

cc, cubic centime; CI, confidence interval; CRLM, colorectal liver metastasis; HCC, hepatocellular carcinoma, FLR, future liver remnant; IHCC, intrahepatic cholangiocellular carcinoma; ISGLS, International Study Group of Liver Surgery; NET, neuroendocrine tumor; n.r, not reported; PHCC, perihilar cholangiocarcinoma; PVE, portal vein embolization; PVE/HVE, simultaneous portal and hepatic vein embolization; SD, standard deviation; sFLR, standardized future liver remnant.

In none of the PVE/HVE vs. PVE cohorts, there was a difference in complication rates between the embolization procedures (Table 2) (73–78). The most frequent complications after PVE/HVE were fever and pain, which are known from PVE as signs of the “post-embolization syndrome”. Concerns about liver necrosis due to the simultaneous occlusion of the hepatic venous in- and outflow remained unfounded (80). Only a slight elevation of transaminases was observed and histological signs of necrosis were comparable to PVE (26, 73). Just as described in the pig model of double ligation (67), it appears that arterial blood flow keeps the embolized liver lobe viable and new venous outflow collaterals from the deportalized side to the growing liver allow venous drainage of the embolized lobe.

**Figure 3:**
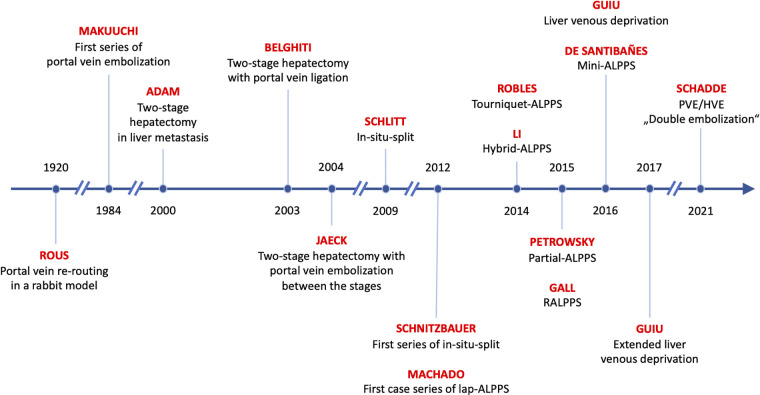
Kinetic growth rate. Volume increase in standardized future liver remnant (sFLR) per week for simultaneous portal and hepatic vein embolization (PVE/HVE) and portal vein embolization (PVE) for *all patients* (**A**) and for *matched subgroups* (**B**), based on a 1:1 match for the closest time to first imaging, age, Charlson comorbidity index, cirrhosis, diabetes, and Bevacizumab. The colored arrows show *median* liver growth for PVE/HVE (orange) and PVE (gray).

While a comparison of liver volume growth in the cohorts remains difficult due to the use of different liver growth units and metrics, all series demonstrate an increased liver growth after PVE/HVE over PVE alone (Table 2) (73–78). In the largest series so far, the cohort study of the DRAGON collaborative (78), liver volume gain was investigated in standardized metrics and revealed a kinetic growth rate (KGR) of 3.5% sFLR/week after PVE/HVE vs. 2.5% sFLR/week (*p* < 0.001) after PVE (Figure 3A). Since patients obtained volumetric assessment sooner after PVE/HVE in DRAGON, an additional sensitivity analysis was performed with growth metric after similar waiting times of 17 (PVE/HVE) and 21 days (PVE), respectively (Figure 3B). KGR remained higher after PVE/HVE (PVE/HVE: 3.5% sFLR/week vs. PVE: 2.7% sFLR/week, *p* = 0.03). While volumetric and functional measurements were not congruent in ALPPS (44), two series by Guiu et al. provided functional assessment by HBS and revealed a strictly congruent increase of volume and function after PVE/HVE (66, 77).

Most importantly, the DRAGON collaborative reported that patients undergoing PVE/HVE had a higher chance to achieve curative liver resection (*p* = 0.007) while time to resection was not different between PVE/HVE and PVE (PVE/HVE: 37 days (IQR 21–52) vs. PVE: 41 days (IQR 28–61), *p* = 0.132) (78).

No study found a difference in complications after liver resection between PVE/HVE and PVE so far (Table 2) (73–78). The incidence of PHLF after PVE/HVE was comparable, and both 30- and 90-day mortality were comparable. Currently, there is no evidence that PVE/HVE is not safe in hepatobiliary malignancies.

Recently, a cohort study evaluated PVE/HVE against ALPPS (79). While percent hypertrophy was comparable, KGR was greater after ALPPS (PVE/HVE: KGR: 2% FLR/day (range 0, 11) vs. ALPPS: 7% FLR/day (range −1, 27), *p* < 0.001). This volumetric assessment, however, is biased since it was performed 8 days (range 1, 43) after ALPPS and 28 days (range 4, 52) after PVE/HVE (*p* < 0.001), which does not allow to evaluate KGR where time is in the denominator. Resectability in PVE/HVE was lower (PVE/HVE: 73% vs. ALPPS: 91%, *p* < 0.001). No differences were seen in terms of post-operative complications and 90-day mortality. However, in PVE/HVE, surgery was performed later, patients were older (*p* = 0.02), and there were more hepatobiliary malignancies (*p* < 0.001) compared with ALPPS.

## What are the alternatives to regenerative liver surgery?

### Parenchymal sparing surgery

Resection of tumor lesions in a parenchymal sparing fashion (parenchymal sparing surgery, PSS), however, allows the removal of multiple tumor lesions without the need for an extended liver resection (81). The Humanitas group in Milan developed the intraoperative ultrasonography (IOUS) criteria as guidelines for the necessity of the resection of tumor-infiltrated portal venous structures and the respective liver parenchyma. When (1) the tumor is separated by a thin layer of parenchyma, (2) the vessel wall shows no discontinuation, and when (3) the tumor surrounds less than 1/3 of the vessel diameter, the tumor should be resected by preserving the portal venous structures and corresponding liver parenchyma, even if a positive histological resection margin is expected. Additionally, IOUS allows the detection of hepatic vein collaterals, which enable the preservation of liver parenchyma despite the resection of the respective hepatic vein. PSS was compared with TSH in a retrospective comparative series of patients with CRLM and oligometastases (82). While blood loss and major complication were less in PSS, complete histological tumor resection (R0) was the same between both approaches. The overall survival was also comparable between both approaches, but patients who failed to achieve the second stage in the TSH group (40%) were not included in the survival analyses.

Despite the need to evaluate every case of a planned TSH for a parenchymal sparing option and the necessity to remind even technically adept and experienced liver surgeons that parenchymal sparing resections can replace a planned TSH, there are two compelling reasons why PSS is not an option: First, when lesions are not just abutting but have a more than 180° involvement of the right or the left inflow pedicle or the three outflow veins and anatomic resection have to be performed: Chemotherapy can sometimes effect a secondary detachment of metastases from vessels due to an intrinsic proliferation of liver tissue that may push itself between a lesion that is shrinking due to chemotherapy and the respective vessel. This phenomenon of chemotherapy-induced tumor detachment, unique to the liver, is generally not observed in other parenchymal organs and rarely with tumors that have more than 180° vessel involvement. A second reason why PSS may not be an option is a diffuse involvement of one liver lobe that cannot be targeted with PSS, because lesions are too close to one another, too multiple, and too deep to be resected in cluster resections.

Due to these very different anatomic scenarios that lead experienced surgeons to choose one approach over the other, a comparative or even randomized trial may not be possible: Situations in which both PSS and TSH are possible and equally helpful are very rare. An unbiased comparison, therefore, is impossible and a randomization ethically not defendable.

### Locoregional heat ablation

In order to avoid the need for a two-stage procedure with the risk of tumor progression while awaiting sufficient liver growth, the MD Anderson group retrospectively evaluated one-stage resection + ablation against classic TSH in patients with CRLM (83). The study revealed an increased major complication rate (resection + ablation: 20% vs. TSH: 6%, *p* < 0.001) and worse 5-year overall survival rate following one-stage resection + ablation (resection + ablation: 24% vs. TSH: 35%, *p* = 0.016). Inversely, the Paul-Brousse group implemented ablation in the concept of one- and two-stage resections in CRLM when the FLR was affected by tumor lesions and showed in a retrospective study comparable post-operative (Dindo–Clavien > III: resection + ablation: 22% vs. resection alone: 19%, *p* = 0.66) and long-term outcomes (5-year survival rate: resection + ablation: 58 months vs. resection alone: 56 months, *p* = 0.57) compared with patients undergoing one- and two-stage resections without ablation (84).

There are some prospective randomized trials comparing resection against ablative procedures in HCC (85–87); however, the results are partially controversial. According to the Barcelona Clinic Liver Cancer (BCLC) criteria, ablation should be considered in the very early (single lesion with less than 2 cm in size and preserved liver function) and early stage (maximum of three lesions with a maximum of 3 cm in size and preserved liver function) when patients are not suitable for surgery (88). Ablation as a fall-back for patients who are not candidates for surgery is non-controversial.

## What is the role of preoperative chemotherapy?

Only 10%–20% of patients with CRLM are primarily resectable (89). In the remaining patients, secondary resectability can eventually be achieved through chemotherapy by downsizing the tumor, detaching tumors from vascular structures and the FLR. However, “conversion-type” preoperative chemotherapy has to be limited, because liver metastasis with a complete remission (no longer radiologically detectable) reappears in 80% after chemotherapy is stopped or becomes ineffective (90). Conversion chemotherapies for CRLM have been tested in the randomized CELIM (91) (FOLFOX + cetuximab vs. FOLFIRI + cetuximab) and OLIVIA (92) (FOLFOXIRI + bevacizumab vs. FOLFOX + bevacizumab) trials accomplishing a complete tumor resection (R0) in 34% and 49% (FOLFOXIRI + bevacizumab), respectively. Of course, survival rates in patients requiring conversion chemotherapy are worse than in patients who are primarily resectable, but better than in patients receiving chemotherapy alone (93).

A recent RCT compared resection + adjuvant chemotherapy (mFOLFOX6) vs. resection and showed that the addition of chemotherapy improves disease free survival, but not survival (94). However, it should be noted that in both groups, patients had a low tumor burden with a maximum of three liver metastases and were therefore not representative for patients with the need for regenerative liver surgery. It is highly unlikely that the conclusions of this trial can be applied to scenarios of initial un- or borderline resectability. In contrast, it is likely that methods like PVE/HVE that reduce the surgical severity of liver metastasis removal but induce fast hypertrophy are advantageous compared with two-stage approaches because they allow patients to remain on chemotherapy until their extensive tumor load is removed. Embolizations can be performed while patients are under chemotherapy, and in many cases of PVE/HVE, only one surgical resection is necessary.

Two RCTs are available in patients with unresectable bile tract cancers, which suggests a moderate conversion rate after chemotherapy (95, 96). The first series comparing gemcitabine + cisplatin vs. gemcitabine alone provided a conversion rate of approximately 20% for both regimes (95). In the second trial, nab-paclitaxel was additionally given to gemcitabine + cisplatin, resulting in a comparable conversion rate of 20% (96). Both trials did not provide further information about the outcome of patients who ultimately underwent surgery and how they were resected.

Whether HCC can be effectively downsized and converted to resectability with the current first-line treatment Atezolizumab-Bevacizumab, Lenvatinib, and Nivolumab remains unclear, but data on this are expected in the near future.

## Conclusion: What is the gold standard of regenerative liver surgery?

In summary, based on long experience and established safety, PVE remains the gold standard of regenerative liver surgery. However, because of limited hypertrophy and therefore resectability, this gold standard will be challenged. ALPPS failed to convince, largely because it is a regenerative strategy tied to a TSH. PVE/HVE, however, is not tied to two stages of surgery and induces rapid liver growth with a safety profile similar to PVE but with the feasibility of ALPPS. The question whether ALPPS or PVE/HVE should replace PVE as the gold standard of regenerative liver surgery has to be answered in multicentre RCTs, two of which are currently underway (DRAGON international: NCT04272931, LVD France: NCT03995459).

## Author contributions

JH conceptualized and designed the study and drafted the manuscript. ES also conceptualized and designed the study and performed a critical revision of the manuscript for important intellectual content. All authors contributed to the article and approved the submitted version.

## Conflict of interest

The authors declare that the research was conducted in the absence of any commercial or financial relationships that could be construed as a potential conflict of interest.
